# Transmission of multidrug-resistant tuberculosis in Jiangxi, China, and associated risk factors

**DOI:** 10.1128/spectrum.03555-23

**Published:** 2024-10-02

**Authors:** Jiahuan Zhan, Wei Wang, Dong Luo, Qiang Chen, Shengming Yu, Liang Yan, Kaisen Chen

**Affiliations:** 1 Department of Clinical Laboratory, The First Affiliated Hospital, Jiangxi Medical College, Nanchang University, Nanchang, China; 2 School of Public Health, Jiangxi Medical College, Nanchang University, Nanchang, China; 3 Department of Clinical Laboratory, Jiangxi Provincial Chest Hospital, Nanchang, China; Foundation for Innovative New Diagnostics, Geneve, Switzerland

**Keywords:** multidrug-resistant tuberculosis, transmission, whole-genome sequencing, risk factor

## Abstract

In order to effectively combat the urgent threat of multidrug-resistant tuberculosis (MDR-TB), it is imperative to gain a comprehensive understanding of the drug-resistant profiles, transmission dynamics, and associated risk factors. Our study encompassed a population-based retrospective analysis with 130 MDR-TB patients from 2018 to 2021. The research methodology incorporated whole-genome sequencing, drug susceptibility testing , and logistic regression analysis to discern the risk factors of genomic clustering linked to recent transmission. The findings from phenotypic drug resistance assessments revealed notable resistance rates: ethambutol at 62.3% (81/130), streptomycin at 72.3% (94/130), levofloxacin at 51.5% (67/130), and moxifloxacin at 50.0% (65/130). Furthermore, among all patients, 38 individuals (29.23%, 38/130) were found to be part of 17 clusters, indicating instances of recent MDR-TB transmission. The genomic clustering patients were deeply investigated. Lineage 2.2.1 was established as the primary sub-lineage (86.15%, 112/130), followed by lineage 4 (9.23%, 12/130). Moreover, the logistic regression analysis underscored that unemployment, farming occupations, and prior TB treatment were identified as significant risk factors for recent transmission.

**IMPORTANCE:**

The high prevalence of multidrug-resistant tuberculosis (MDR-TB) in Jiangxi Province highlights the importance of understanding the genetic background and drug resistance patterns of these strains. This knowledge is crucial for developing effective control methods. Furthermore, in light of the significance of preventing transmission among tuberculosis patients, whole-genome sequencing was utilized to investigate the recent transmission of MDR-TB and identify associated risk factors. The findings revealed that individuals in the farming sector, those who are unemployed, and patients with a history of tuberculosis treatment are at elevated risk. Consequently, targeted public interventions for these at-risk groups are imperative.

## INTRODUCTION

The burden of tuberculosis (TB) remains a significant public health concern. Despite the dominance of COVID-19 as the primary epidemic in 2021, the 2023 World Health Organization TB report has brought attention to the alarming figures of 10.6 million new TB cases and 1.3 million deaths. This surpasses other infectious diseases, including AIDS (1). It is crucial to acknowledge tuberculosis as a major infectious disease. Drug-resistant tuberculosis (DR-TB), particularly multidrug-resistant tuberculosis (MDR-TB), has garnered considerable attention from healthcare professionals and researchers involved in tuberculosis prevention and control. This is primarily due to its prolonged treatment duration, high treatment costs, strong potential for transmission, and high prevalence (2). In 2022, the global number of MDR/RR-TB patients was estimated at around 0.41 million. The estimated proportion of individuals with MDR/RR-TB was 3.3% among new cases and 17% among those previously treated. These statistics pose a significant challenge for TB control (1).

China is ranked third highest globally in terms of TB burden, with an estimated 0.75 million new cases reported annually (1). Despite this, China has made notable progress in TB control, achieving a total reduction of 55.4% from 1990 to 2019 and meeting the United Nations TB control goals 5 years ahead of the deadline (3). However, the considerable population size and regional variances in TB incidence present challenges for disease control efforts in highly endemic areas of China (4).

In Jiangxi Province, located in the southeast region of China, the incidence rate of MDR-TB is considerably high, which could be attributed to a shortage of professional personnel, inadequate financial support, or patients’ poor adherence to the directly observed treatment short-course regimen (5). Previous single-center epidemiological surveys have indicated that the prevalence of MDR-TB in Jiangxi Province is 14.7%, exceeding the reported levels of MDR-TB in both China (6.9%) and worldwide (3.3%) (1, 6, 7). It is crucial to comprehend the underlying factors contributing to this elevated MDR-TB prevalence to formulate effective TB control strategies. Exogenous infections frequently occur due to interpopulation transmission. Control measures should involve actively identifying the source of infection and promptly isolating and treating TB patients (8).

Molecular typing technology, particularly whole-genome sequencing (WGS), is instrumental in the identification of exogenous infections or endogenous relapses. Strains of the same type are generally indicative of a recent transmission connection (9). Whole-genome sequencing has been widely used in recent years to investigate the molecular epidemiology of TB. The methodology entails the procurement of tuberculosis bacteria from a specific geographic area and time period, the scrutiny of polymorphism in conserved nucleotide sequences across diverse strains, and the determination of strain identity based on the conventional mutation frequency of strains (10). This approach is employed to define clusters, with strains within the same cluster commonly considered to be the result of transmission (11, 12). To investigate the factors contributing to the high prevalence of MDR-TB in Jiangxi Province, a collection of MDR-TB bacteria was obtained from a designated tertiary tuberculosis hospital in Jiangxi Province between 2018 and 2021. Subsequently, extensive genomic epidemiological investigations were undertaken to analyze the drug-resistant profile and transmission dynamics of the MDR-TB strains. These efforts have yielded valuable epidemiological insights that can be instrumental in the control of MDR-TB.

## RESULTS

### Demographic and clinical features

During the study period, a total of 9,579 pulmonary tuberculosis patients underwent treatment, with 4,217 patients testing positive for tuberculosis through culture results. Among the positive cases, 130 patients were identified as suffering from MDR-TB. Of the MDR-TB patients, 71.54% (93/130) were male, 53.85% (70/130) were either farmers or the unemployed, and 63.08% (82/130) had only received junior high school education or below. Furthermore, 23 patients had comorbid diabetes, and 78 patients (60%) had a history of previous tuberculosis treatment (as shown in Table 1). A comparison with 568 contemporaneous DR-TB patients revealed that individuals who were farmers, had junior high school education or below, demonstrated lung cavitation, malnutrition, and had previously undergone tuberculosis treatment were more likely to develop MDR-TB. Detailed demographic and clinic characteristics of patients are presented in Table 1.

**TABLE 1:** Demographic and clinical features of patients with MDR-TB^*a*^

Characteristics	Non-MDR-TB (*n* = 568)		MDR-TB patients (*n* = 130)		*P* value	OR (95% CI)
	No.	%	No.	%		
Sex						
Female	160	28.17	37	28.46		Ref.
Male	408	71.83	93	71.54	0.95	0.99 (0.65–1.50)
Age (years)						
＜0	166	29.23	32	24.62		Ref.
30–44	112	19.72	28	21.54	0.36	1.30 (0.74–2.73)
45–59	128	22.54	37	28.46	0.13	1.50 (0.89–2.54)
≥60	162	28.52	33	25.38	0.84	1.06 (0.62–1.80)
BMI						
Low (<18.5）	200	35.21	59	45.38	0.99	1.00 (0.48–2.08)
Normal (18.5–24）	316	55.63	61	46.92		Ref.
High (>24）	52	9.15	10	7.69	0.26	1.53 (0.74–3.20)
Marriage						
Unmarried	144	25.35	26	20.00		Ref.
Married	424	74.65	104	80.00	0.20	1.36 (0.85–2.17)
Occupation						
Others	322	56.69	60	46.15		Ref.
Farmer	204	35.92	62	47.69	0.02	1.63 (1.10–2.42)
Unemployed	42	7.39	8	6.15	0.96	1.02 (0.46–2.29)
Educational attainment						
High school and above	280	49.30	48	36.92		Ref.
Junior high school and below	288	50.70	82	63.08	0.01	1.66 (1.12–2.46)
Lung cavitation						
No	254	44.72	42	36.92		Ref.
Yes	314	55.28	88	63.08	0.01	1.70 (1.13–22.54)
Underlying medical conditions						
No	356	62.68	80	61.54		Ref.
Diabetes	110	19.37	23	17.69	0.78	0.93 (0.56–1.55)
Others	102	17.96	27	20.77	0.51	1.18 (0.72–1.92)
Smoking						
No	424	74.65	92	70.77		Ref.
Yes	144	25.35	38	29.23	0.36	1.22 (0.80–1.86)
Drinking						
No	494	86.97	119	91.54		Ref.
Yes	74	13.03	11	8.46	0.15	0.62 (0.32–1.20)
Diagnosis delay						
No	298	52.46	61	46.92		Ref.
Yes	270	47.54	69	53.08	0.26	1.25 (0.85–1.23)
Nutritional status						
Good	274	48.24	28	21.54		Ref.
Medium	190	33.45	61	46.92	<0.001	3.14 (1.94–5.10)
Malnutrition	104	18.31	41	31.54	<0.001	3.86 (2.27–6.56)
Previous TB treatment						
No	466	82.04	52	40.00		Ref.
Yes	102	17.96	78	60.00	<0.001	6.85 (4.54–10.34)

^*a*^ Ref., reference.

### Patterns of drug resistance patterns

The resistance rates to streptomycin (SM) and ethambutol (EMB) among the 130 MDR-TB strains were found to be 72.3% (94/130) and 62.3% (81/130), respectively, based on the phenotypic susceptibility profiles of 52 treatment-naïve tuberculosis patients and 78 retreated patients. Notably, the resistance rates to two fluoroquinolones (FQs), levofloxacin (LEV) and moxifloxacin (MFX), were even higher, with LEV at 51.5% and MFX at 50.0% among the second-line anti-tuberculosis drugs (Table 2).

**TABLE 2:** Drug-resistant profiles of 130 MDR-TB strains^*a*^

Type of resistance	No.		% (95% CI)	
	New patients	Retreated patients	New patients	Retreated patients
Isoniazid	49	81	37.69% (29.25%–46.13%)	62.31% (53.87%–70.75%)
Rifampicin	49	81	37.69% (29.25%–46.13%)	62.31% (53.87%–70.75%)
Ethambutol	35	46	29.92% (19.20%–34.65%)	35.38% (27.06%–43.71%)
Streptomycin	39	55	30.00% (22.02%–37.98%)	42.31% (33.70%–50.91%)
Amikacin	11	10	8.46% (3.61%–13.31%)	7.69% (3.05%–12.33%)
Prothionamide	12	17	9.23% (4.19%–14.27%)	13.08% (7.20%–18.95%)
Capreomycin	8	10	6.15% (1.97%–10.34%)	7.69% (3.05%–12.33%)
Levofloxacin	26	41	20.00% (13.03%–26.97%)	31.54% (23.44%–39.63%)
Cycloserine	2	1	1.54% (0.00%–3.68%)	0.77% (0.00%–2.29%)
Moxifloxacin	22	43	16.92% (10.39%–23.45%)	33.08% (24.88%–41.27%)
Pre-XDR-TB^*b*^	20	41	15.38% (9.10%–21.67%)	31.54% (23.44%–39.63%)

^*a*^ Since all strains are sensitive to clarithromycin, the susceptibility information for clarithromycin is not included.

^*b*^ XDR-TB, extensively drug-resistant tuberculosis.

### Characterization of drug resistance mutation spectrum of MDR-TB

In our analysis, it was found that rifampicin (RIF) displayed the highest percentage of molecular resistance at 95.38% (124 out of 130), with the most prevalent mutation type being *rpoB* S450L, accounting for 59.68% of the mutant strains, followed by *rpoB* L430P at 6.45%. Isoniazid (INH) showed a resistance percentage of 88.46%, primarily associated with the mutation type *katG* S315T, which contributed to 77.93% (89 out of 115). The resistance percentages for EMB and SM were 60% and 68.46%, respectively, with the most common mutation type for EMB and SM being *embB* M306V and *rpsL* K43R, respectively. FQs exhibited a resistance percentage of 50.77%, with the most prevalent type of mutation being *gyrA* D94G. Amikacin (AK), capreomycin (CM), and kanamycin (KM) displayed resistance percentages of 16.92%, 16.15%, and 19.23%, respectively. The mutation type *rrs* A1401G was found to be prevalent in AK, CM, and KM (as shown in Table 3). It is critical to identify the specific mutations and understand their impact on drug efficacy, as various mutation types contribute to drug resistance. Genotypic resistance was determined when drug resistance mutations were present, while genotypic susceptibility was concluded in the absence of such mutations, using phenotypic drug sensitivity results as a reference standard. The phenotypic drug susceptibility testing results indicated that the sensitivity of WGS demonstrated a sensitivity of 90% or higher in detecting resistance to RIF, SM, AK, CM, and MFX as well as a sensitivity of over 83% for INH, EMB, and LEV; the agreement between the two methods in determining strain resistance was observed in more than 85% of strains for all drugs (Table 4).

**TABLE 3:** Drug resistance gene mutation spectrum of 130 MDR-MTB strains

Drug	Number of drug-resistant strains (percentage)	Mutation type	Number of mutant strains	Mutation percentage
Rifampicin	124 (95.38%)	rpoB_p.S450L	74	59.68%
		rpoB_p.L430P	8	6.45%
		Others	42	33.87%
Isoniazid	115 (88.46%)	katG_p.S315T	89	77.39%
		inhA_c.-777C > T	15	13.04%
		Others	11	9.57%
Ethambutol	78 (60.00%)	embB_p.M306V	39	50.00%
		embB_p.M306I	12	15.38%
		Others	27	34.62%
Streptomycin	89 (68.46%)	rpsL_p.K43R	73	82.02%
		Others	16	17.98%
Fluoroquinolones	66 (50.77%)	gyrA_p.D94G	31	46.97%
		gyrA_p.A90V	24	36.36%
		Others	11	16.67%
Amikacin	22 (16.92%)	rrs_n.1401A > G	21	95.45%
Capreomycin	21 (16.15%)		21	100.00%
Kanamycin	25 (19.23%)		21	84.00%
		Others	4	16.00%

**TABLE 4:** Comparison of phenotypic and genotypic resistance of 130 strains of MDR-TB

Drug	Genotypic drug	Phenotypic drug sensitivity		Concordance (%)	Positive	Negative
	Sensitivity	R	S		Predictive value	Predictive value
Rifampicin	R	124	0	95.38%	100.00%	NA^*a*^
	S	6	0			
Isoniazid	R	115	0	88.46%	100.00%	NA^*a*^
	S	15	0			
Ethambutol	R	70	8	85.38%	89.74%	78.85%
	S	11	41		(81.05%–94.71%)	(65.97%–87.76%)
Streptomycin	R	88	1	94.62%	98.88%	85.37%
	S	6	35		(93.91%–99.80%)	(71.56%–93.12%)
Levofloxacin	R	59	7	88.46%	89.39%	87.50%
	S	8	56		(79.69%–94.77%)	(77.23%–93.53%)
Amikacin	R	19	3	96.15%	86.36%	98.15%
	S	2	106		(66.67%–95.25%)	(93.50%–99.49%)
Capreomycin	R	15	6	93.08%	71.43%	97.25%
	S	3	106		(50.04%–86.19%)	(92.22%–99.06%)
Moxifloxacin	R	62	4	94.62%	93.94%	95.31%
	S	3	61		(85.43%–97.62%)	(87.10%–98.39%)

^*a*^ NA, not applicable.

### Phylogenetic study

**Fig 1:**
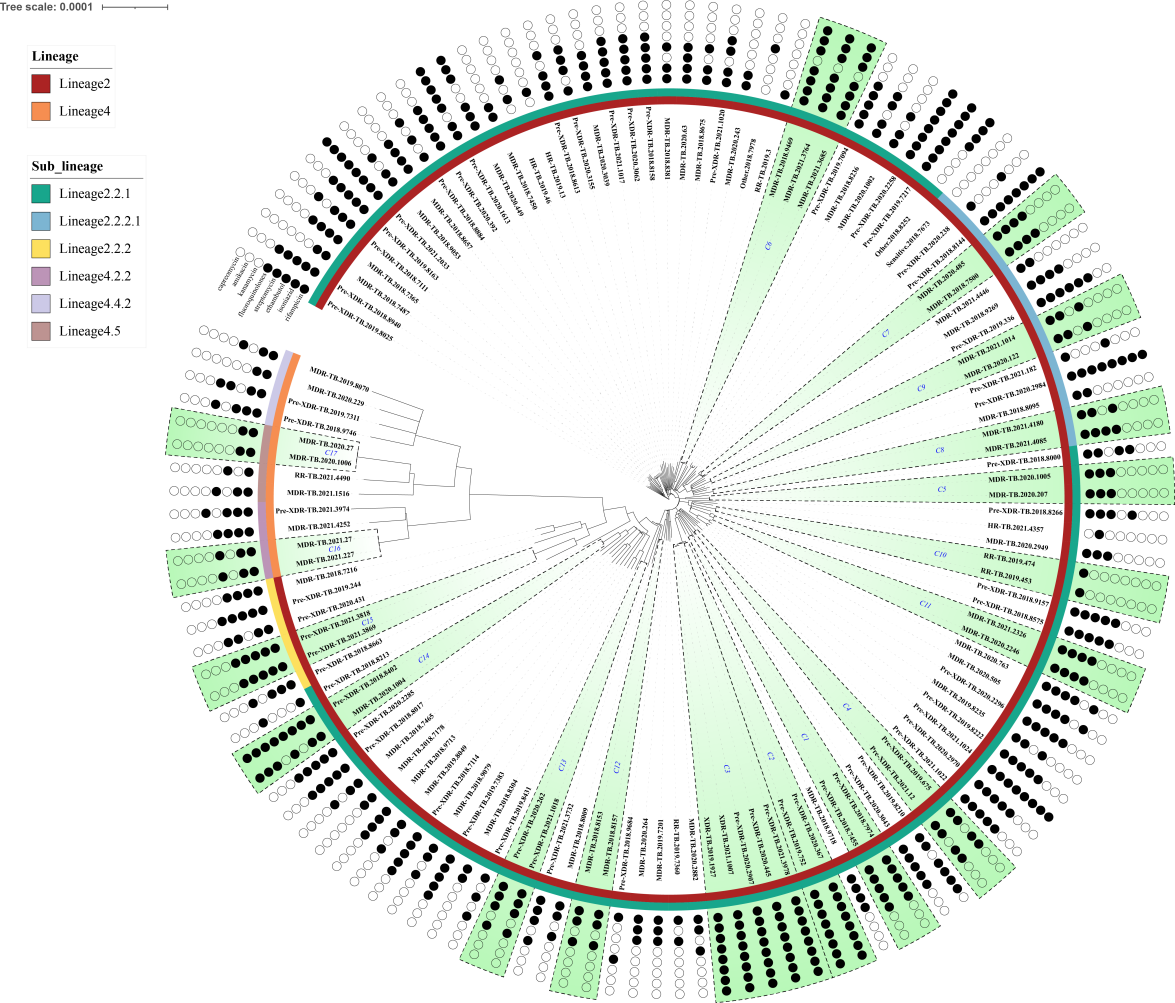
Maximum likelihood tree constructed using 130 multidrug-resistant tuberculosis strains, including annotation detailing the drug resistance profiles of the strains. The tree utilizes color-coded indicators to represent lineage and genotypic resistance spectrum. Genotype resistance to eight anti-tuberculosis drugs is indicated by black or white circles denoting resistance and sensitivity, respectively. Strain notes: phenotypic resistance type, year of origin, and sampling number.

A total of 130 patient specimens diagnosed with MDR-TB between 2018 and 2021 were selected for this study following strain culture, drug sensitivity testing, and whole-genome sequencing. The sequencing data were filtered using specific parameters (median coverage depth >10×, coverage breadth >90%, major strain frequency >80%, and nontuberculous mycobacteria frequency <20%). An evolutionary tree was constructed based on the whole-genome sequencing data to comprehend the genetic background and to evaluate the recent transmission patterns of these strains. The analysis revealed two primary lineages: 90.77% (118/130) of MDR-TB strains were attributed to lineage 2 (East Asian genotype), while the remaining 9.23% (12/130) belonged to lineage 4. Within lineage 2, the dominant sub-lineage was lineage 2.2.1 (Beijing genotype), representing 86.15% (112/130) of the strains, while lineage 2.2.2 encompassed six strains (Fig. 1).

**Fig 2:**
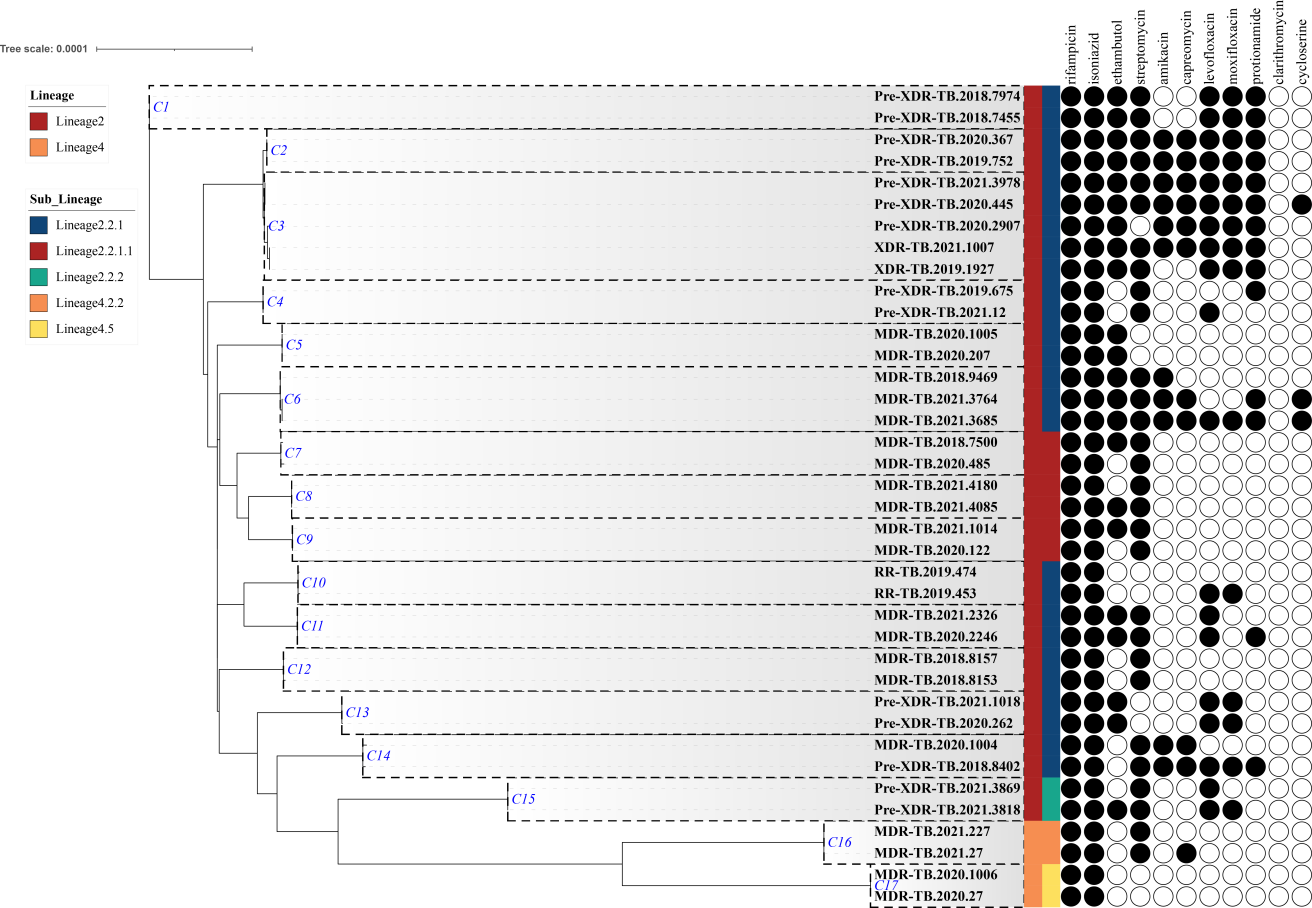
Maximum likelihood tree and phenotypic resistance spectra of 38 rifampicin-resistant tuberculosis strains categorized into 17 groups. Each cluster, labeled C1–C17, is delineated by a dotted line on the graph. The genotype resistance to seven anti-tuberculosis drugs is denoted by black and white circles, where black circles signify resistance, and white circles indicate sensitivity.

Based on the WGS data and using a cutoff of 12 single-nucleotide polymorphisms (SNPs) for defining genomic transmission clusters, a total of 38 strains were identified, encompassing 17 clusters. Among these, 15 clusters were attributed to the Beijing family, while two clusters were associated with a non-Beijing family. Cluster sizes varied from two to five cases, with cluster 3 (C3) comprising five cases and cluster 6 (C6) containing three cases. The remaining clusters consisted of two cases each (Fig. 2).

### Risk factors of genomic clustering

A statistical analysis was performed to identify the risk factors associated with the genomic clusters of MDR-TB. The results revealed that farmers (OR = 2.58, *P* = 0.034) and the unemployed (OR = 7.14, *P* = 0.018) exhibited a higher likelihood of contributing to genomic clusters compared to individuals in other occupations. Furthermore, retreated patients were closely associated with the recent spread of MDR-TB (OR = 2.96, *P* = 0.019). However, the differences between other factors and the spread of MDR-TB did not demonstrate statistical significance (as shown in Tables 5 and 6).

**TABLE 5:** Univariate analysis of the risk factor for recent transmission among 130 MDR-TB^*a*^

Characteristics	Non-cluster (*n* = 92)		Cluster (*n* = 38)		*P* value	OR (95% CI)
	No.	%	No.	%		
Sex						
Female	25	27.17	12	31.58		Ref.
Male	67	72.83	26	68.42	0.61	0.81 (0.36–1.84)
Age (years)						
<30	22	23.91	10	26.32		Ref.
30–44	18	19.57	10	26.32	0.72	1.22 (0.42–3.58)
45–59	27	29.35	10	26.32	0.70	0.82 (0.29–2.31)
≥60	25	27.17	8	21.05	0.53	0.70 (0.24–2.10)
BMI						
Low (<18.5）	45	45.65	14	36.84	0.36	0.69 (0.31–1.54)
Normal (18.5–24）	42	48.91	19	50.00		Ref.
High (>24）	5	5.43	5	13.16	0.25	2.21 (0.57–8.55)
Marriage						
Unmarried	16	17.39	10	26.32		Ref.
Married	76	82.61	28	73.68	0.25	0.59 (0.24–1.45)
Occupation						
Others	50	54.35	10	26.32		Ref.
Farmer	39	42.39	23	60.53	0.01	2.95 (1.26–6.92）
Unemployed	3	3.26	5	13.16	0.01	8.33 (1.71–40.63)
Educational attainment						
High school and above	32	34.78	16	42.11		Ref.
Junior high school and below	60	65.22	22	57.89	0.43	0.73 (0.34–1.59)
Lung cavitation						
No	26	28.26	16	42.11		Ref.
Yes	66	71.74	22	57.89	0.13	0.54 (0.25–1.19)
Underlying medical conditions						
No	57	61.96	23	60.53		Ref.
Diabetes	17	18.48	6	15.79	0.80	0.88 (0.31–2.50)
Others	18	19.57	9	23.68	0.65	1.24 (0.49–3.16)
Smoking						
No	67	72.83	25	65.79		Ref.
Yes	25	27.17	13	34.21	0.42	1.39 (0.62–3.14)
Drinking						
No	86	93.48	33	86.84		Ref.
Yes	6	6.52	5	13.16	0.23	2.17 (0.62–7.60)
Diagnosis delay						
No	45	48.91	16	42.11		Ref.
Yes	47	51.09	22	57.89	0.48	1.32 (0.61–2.82）
Nutritional status						
Good	17	18.48	11	28.95		Ref.
Medium	44	47.83	17	44.74	0.28	0.60 (0.23–1.53)
Malnutrition	31	33.70	10	26.32	0.19	0.50 (0.18–1.41)
Previous TB treatment						
No	44	47.83	8	21.05		Ref.
Yes	48	52.17	30	78.98	0.01	3.44 (1.43–8.29）
Lineages						
Lineage 2	84	91.30	34	89.47		Ref.
Lineage 4	8	8.70	4	10.53	0.74	1.24 (0.35–4.38)
DR type						
Others	45	48.91	22	57.89		Ref.
Pre-XDR-TB/XDR-TB^*b*^	47	51.09	16	42.11	0.35	0.70 (0.33–1.49)

^*a*^ OR, odds ratio; Ref., reference.

^*b*^ XDR-TB, extensively drug-resistant tuberculosis.

**TABLE 6:** Multivariate logistic regression of the risk factor for recent transmission among 130 MDR-TB^*a*^

Characteristics	AOR (95% CI)	*P* value
Occupation		
Others	Ref.	
Farmer	2.58 (1.08–6.17)	0.034
Unemployed	7.14 (1.40–36.31)	0.018
Previous TB treatment		
No	Ref.	
Yes	2.96 (1.19–7.34)	0.019

^*a*^ AOR, adjusted odds ratio; Ref., reference.

## DISCUSSION

This study represents the initial exploration of the prevalence of MDR-TB utilizing a WGS approach in Jiangxi Province, China. Our analysis revealed that lineage 2 (East Asian genotype) was the predominant lineage, constituting over 90% of the isolates. While prior studies had connected drug resistance to the Beijing genotype (13, 14), our results indicated a weaker association between the Beijing genotype and drug resistance (15). This disparity could be attributed to differences in the quantity, origin, and geographical distribution of the collected strains. While certain studies have demonstrated that lineage 2 displays higher transmissibility (16), we did not observe a significant correlation between genotype and clustering rates of MDR-TB strains (clustered L2/4 = 34/4, non-clustered L2/4 = 84/8). This outcome could be influenced by variations in sample sources, study periods, and the use of different SNP thresholds for defining clusters.

In our study, it was observed that more than 40% of cases of MDR-TB had progressed to pre-extensively drug-resistant tuberculosis (pre-XDR-TB). The findings revealed that 72.3% of MDR-TB strains exhibited resistance to SM, aligning with similar reports from Guangdong (68.35%) (17) and Zambia (65.9%) (18). Notably, the resistance to EMB among MDR-TB cases was significantly high at 62.3%, falling below the results from Bangladesh (80%) (19) but exceeding those from Dalian (19.5%) (20) and Thailand (52.84%) (21). The heightened EMB resistance holds critical relevance as it is a primary first-line drug in the treatment of TB, thereby limiting therapeutic options due to increased resistance levels (22). Our investigation further disclosed a phenotypic resistance rate of over 50% for second-line anti-tuberculosis drugs, particularly fluoroquinolones, signaling a concerning scenario with limited restricted treatment alternatives for MDR-TB. This situation is exacerbated by the continued use of EMB and FQs as primary agents in managing MDR and RIF-resistant TB cases (23).

**Fig 3:**
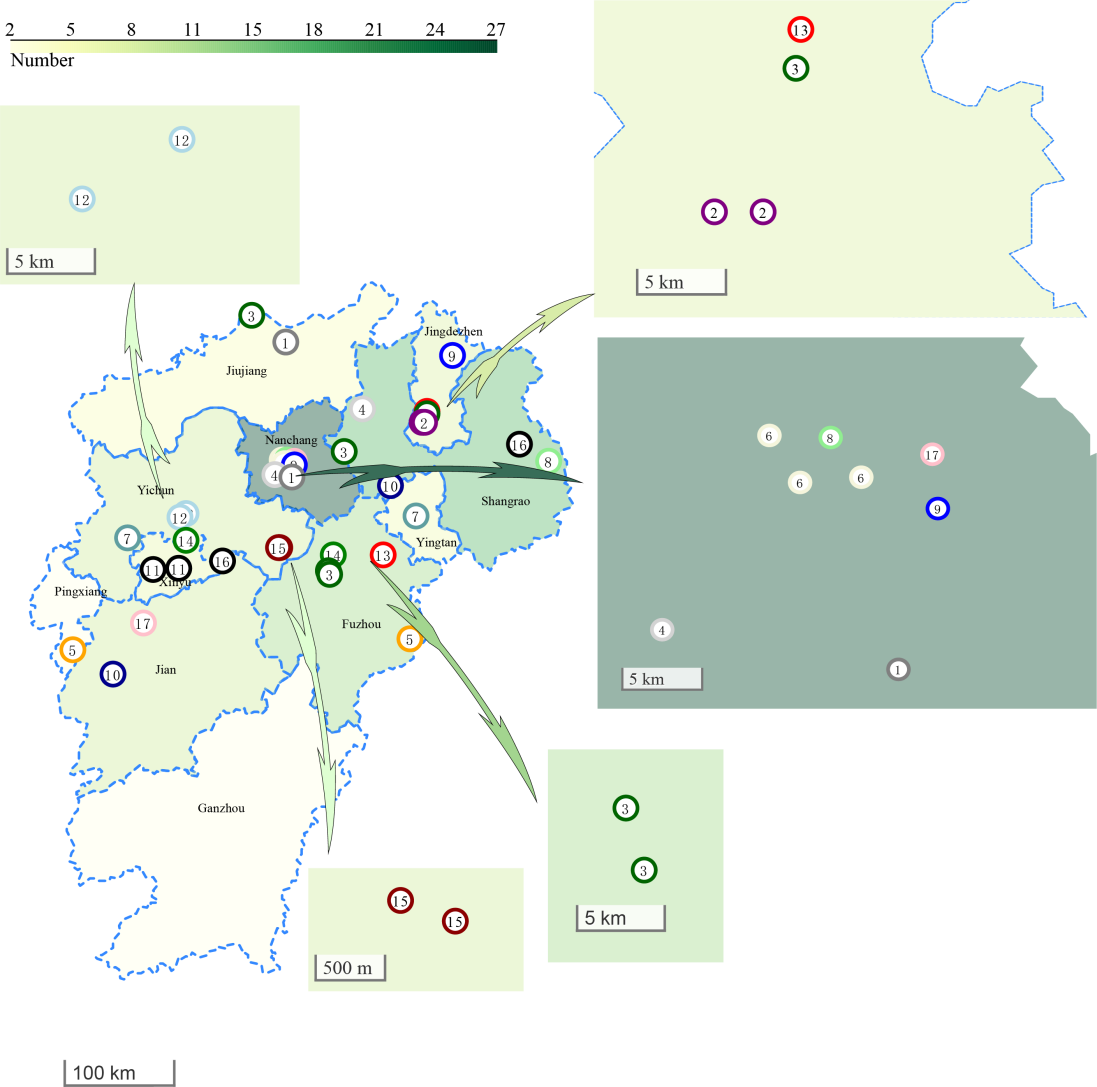
Geographic distribution of clustered strains in Jiangxi Province.

In addition to evaluating phenotypic drug resistance patterns, we conducted an analysis of the molecular mechanism underlying resistance to anti-TB drugs. Through whole-genome data using a TB profiler, we screened gene mutations (24). Consistent with previous research, mutations associated with resistance to rifampicin and isoniazid were primarily localized to the RIF-resistance determining region (RRDR) and the *katG315* coding region (6, 25–27). Since RIF-resistant determinants are primarily concentrated within the 81-bp RRDR, the development of genetic diagnostic techniques can contribute to the diagnosis of RIF resistance in tuberculosis bacteria (28, 29). Our comparison of gene sequencing results for predicting drug resistance with phenotypic drug sensitivity outcomes demonstrated good consistency, aligning with findings from other studies (30). Our investigation encompassed an analysis of both the drug resistance spectrum and the transmission status of MDR-TB. The findings revealed a clustering rate of 29.2%, signifying the substantial role of transmission in the prevalence of MDR-TB in Jiangxi Province. Notably, this rate slightly surpasses the recent transmission of MDR-TB in Beijing, China (24.66%) (31), and falls below that of Chongqing, China (42.8%) (32). With disparately distributed economic development across China, numerous areas within Jiangxi Province lack comprehensive tuberculosis surveys, leading to many patients seeking medical intervention only upon exhibiting typical TB symptoms. Consequently, some patients may remain in the active stage of pulmonary tuberculosis for prolonged periods, thereby facilitating person-to-person transmission. The existing substantial evidence indicates that MDR-TB is primarily the results of recent transmission within the population (33). However, the relatively low transmission rate observed in our study may be attributed to insufficient collection of patient strains. For instance, our traditional epidemiological survey of 38 patients in 17 clusters revealed that only 11 patients in clusters 2, 3, 6, 12, and 15 were geographically close, with a distance ranging from 1.0 to 6.0 km. Furthermore, it was noted that two patients in cluster 2 frequently visited a supermarket for shopping and two patients in cluster 15 in adjacent villages (Fig. 3).

Factors influencing the spread of MDR-TB were studied by analyzing the characteristics of genomic clusters and non-clustered populations. Farmers in China have been identified as a significant risk factor for TB and MDR-TB (34). This is attributed to their role as the primary driving force behind rural-to-urban population migration, a result of China’s reform process, consequently contributing to the spread of tuberculosis (35). The imperfect off-site medical system further exacerbates the situation by causing delays in the diagnosis and treatment of tuberculosis among farmers. Additionally, lower levels of education and limited awareness about tuberculosis make farmers more susceptible to tuberculosis infection (36). These findings are supported by the studies of Meng Li et al. (37) on transmission risk factors in Sichuan and by FAN Yu Feng et al. (38), who investigated the national situation in 2020. Elevating awareness regarding the prevalence of tuberculosis and enhancing access to medical care off-site are crucial measures in mitigating the spread of tuberculosis within the farming community. The lack of employment not only correlates with the occurrence of MDR-TB (39, 40) but also influences its transmission. Unemployment is frequently associated with lower income, signifying a socioeconomic disadvantage, which is a common factor associated with MDR-TB. Our findings indicate that unemployment is also correlated with the recent spread of MDR-TB, likely due to the heightened susceptibility of the unemployed population resulting from their limited access to information, inadequate nutrition, prolonged physical labor, and limited range of activities.

Our study highlights that a previous tuberculosis infection not only increases the risk of MDR-TB infection but also functions as a contributing factor to the recent dissemination of MDR-TB. It underscores the imperative for enhanced initial tuberculosis treatment strategies in Jiangxi Province, including advancements in patient treatment adherence, enhancing patient immunity, and refining the evaluation of therapeutic drugs to preclude the influx of substandard medications into the market. This perspective is corroborated by various studies. For instance, data from Ethiopia spanning from 1997 to 2017 revealed significantly higher MDR-TB infection rates among patients with a history of treatment compared to those with an initial diagnosis (41). The data collected in Shenzhen from 2012 to 2020, a major city in southern China, indicated that individuals with tuberculosis were more likely to acquire MDR-TB (42). Previous tuberculosis elevated the risk of MDR-TB but also acted as a contributing factor to recent transmission. For instance, Nodieva et al. (43) documented that factors leading to the recent spread of MDR-TB in a particular hospital included strains of the Beijing genotype, prior hospitalization, and past tuberculosis treatment. However, Toit et al. (44) claimed that the spread of MDR-TB might not be connected to exposure to tuberculosis but rather to variables such as multidrug resistance, ethnicity, and positive HIV status. As a result, risk factors for MDR-TB transmission may demonstrate variability based on regional and population-specific characteristics.

In the present study, a comprehensive genome-wide sequencing analysis was conducted on MDR-TB strains obtained from the period spanning from 2018 to 2021. Additionally, an analysis of the risk factors for clustered and non-clustered patients was undertaken. However, it is important to note that this study had certain limitations. First, as the collected strains were derived solely from the tertiary hospital in Jiangxi Province, there is a likelihood that the recent transmission and infection rates might have been underestimated. Second, the limited strain collection may have led to inherent deviation in the investigation of risk factors for transmission. Consequently, the generalizability of the study results may be constrained. Lastly, being a retrospective study, data that were absent due to patient mortality or other factors were excluded from the analysis, resulting in potential disparities between the obtained results and the actual circumstances.

The predominant MDR-TB in Jiangxi Province is primarily attributed to Beijing genotype bacteria. It is imperative to ascertain the causative factors and control measures associated with the prevalence of the Beijing genotype. Despite the observed relatively low spread of MDR-TB in recent years in this study, more stringent targeted interventions and screening methods are warranted. This is particularly necessary in light of the limitations in strain collection sources and the challenges associated with selecting drugs for the treatment of MDR-TB.

This limitation arose from the exclusive sourcing of our MDR-TB strains from Jiangxi Provincial Chest Hospital. Although patients with severe tuberculosis and MDR-TB are advised to seek treatment at designated hospitals, some individuals infected with tuberculosis opt for medical care in alternative locations, such as Shanghai. The acquisition of samples from specific patients may present challenges due to concurrent illnesses or mortality. Routine screening of high-risk populations and prompt, effective management of MDR-TB patients can contribute to a reduction in the prevalence of MDR-TB (45).

## MATERIALS AND METHODS

### Inclusion/exclusion criteria

In this study, patients who had been diagnosed with tuberculosis in accordance with the “Criteria for PTB Diagnosis” of China (WS288-2017) were included. The minimum requirement for inclusion in the study was sputum smear or/and GeneXpert MTB/RIF positivity in clinical specimens, which were subsequently isolated and cultured on a Löwenstein-Jensen medium. Exclusion criteria for specimen elimination were limited to inadequate genome quality and unsuccessful genome extraction tests.

### Sample collection

The MDR-TB strains were obtained from a diverse group of patients between 2018 and 2021 at Jiangxi Provincial Chest Hospital. The hospital is the sole tertiary comprehensive tuberculosis facility in Jiangxi Province and is designated for the treatment of tuberculosis patients. The strains were identified using PCR-reverse point hybridization, phenotypic susceptibility testing with specific critical concentration values detailed in Table S1, and WGS techniques. In our retrospective observational study based on population, we collected 130 MDR-TB strains, excluding culture-failed strains and consecutive samples from the same patients.

### Patient information

Variables and demographic information of each MDR-TB case were obtained from the electronic medical record system of Jiangxi Chest Hospital. The data encompassed sociodemographic information, treatment history, and various patient characteristics, including gender, age, body mass index, occupation, education level, home address, history of underlying diseases, previous tuberculosis treatment, and nutritional status. Nutritional status was evaluated by clinicians upon admission using the NRS-2002 scale. Notably, due to privacy regulations, HIV infection was not included in the public medical records.

### Strain identification

In this study, strain identification was performed by PCR-reverse point hybridization. DNA amplification was initiated by centrifuging the PCR reaction tube at 5,000 rpm for 2 s, followed by the addition of 4 µL of DNA extracted from the specimen under study. Two PCR tubes were employed simultaneously, with MSI-positive (Inactivated mycobacterium-positive strain) and MSI-negative (Inactivated *E. coli* strain) control DNA added as quality controls. The reaction mixture was subjected to 30 cycles of amplification (50°C for 2 min, 95°C for 10 min, 95°C for 45 s, and 68°C for 60 s), followed by a 10 min extension at 68°C.

The membrane strip was inserted into a 15-mL centrifuge tube, followed by the addition of 5–6 mL of solution A and all PCR products and through mixing. Subsequently, the centrifuge tubes were subjected to heating at 100°C for 10 min and allowed to hybridize at 59°C for 1.5 h. Simultaneously, MSI-positive and MSI-negative controls were processed in the same process. For the film washing, the membrane strip was removed from the tube and washed in a 50-mL sterile tube for 15 min. For color development, a 1:2,000 POD (Streptavidin-Horseradish Peroxidase) solution was prepared at room temperature. The membrane strip was immersed in the POD solution for 30 min, after which, the POD solution was discarded. The strip was rinsed twice with solution A for 5 min per wash and, then, washed with solution C for 2 min, while the color solution was being prepared. Finally, the film strip was immersed in a light-protected chromogenic solution for 10 min, and the results were observed.

### Phenotypic susceptibility testing

In this study, nine anti-tuberculosis drugs were tested using the rapid drug susceptibility detection technology (BACTEC MGIT 960 system). The protocol involved vortexing 6.0 mL of a bacterial solution with glass beads in a centrifuge tube for 15–30 s, followed by the addition of 800 µL of the solution to 12 7.0 mL BBL MGIT tubes. The first tube served as the control, while tubes 2–12 corresponded to specific drugs: INH, RIF, SM, EMB, AK, CM, LEV, Cs (Cycloserine), and MFX. Next, 100 µL of the corresponding drug was added to the respective tubes, and 50 µL of diluted bacterial suspension was added to the control tube. Furthermore, 500 µL of bacterial solution was added to tubes 2–12, and the turbidity of the bacterial solution was adjusted to 0.5 McFarland standard. The tubes were incubated in the BACTEC MGIT 960 system, which facilitated automatic result interpretation with the capability to retest reported incorrect drug susceptibilities. The quality control strains used were *Mycobacterium tuberculosis* ATCC27294 and H37Rv. PZA (Pyrazinamide) was excluded from the analysis due to incomplete data resulting from the necessity for special media.

### DNA extraction and sequencing

All MDR-TB strains were extracted from the L-J medium, and mycobacterial DNA was prepared using the cetyltrimethylammonium bromide method (46). The sequencing library employed a 2 × 150 bp paired-end sequencing strategy using the HiSeq 2000 sequencing platform (Illumina) to achieve 100× coverage. Whole-genome sequencing was conducted by Novo Bioinformatics Technology Co., Ltd. (Tianjin, China), and the data were obtained for 130 strains.

### Data analysis

Following the completion of WGS for *Mycobacterium tuberculosis*, an initial assessment of the FASTQC data was conducted using the fastQC and fastp software to refine and remove low-quality sequences based on default parameters. The BWA and SAMtools software were then employed to align the sequencing data against the reference sequence of the *Mycobacterium tuberculosis* H37Rv standard strain (NC_000962.3), while PCR repeats were excluded. Subsequently, Freebayes was used to identify population-level variations in the resulting BAM files with rigorous filtering criteria applied to exclude low-quality mutation sites. Variant screening included a minimum coverage depth of at least 10×, a minimum mass fraction of Q20 per variant, and an allele frequency exceeding 75%. Alignment of the SNP sites of the isolates with the reference sequence demonstrated a minimum alignment of 95%, excluding SNPs in genomic repeat regions, such as the PE/PPE-PGRS family genes, insertions, mobile elements, or bacteriophage sequences. The filtered sequencing data were assembled using spades to generate the scaffold sequence. Phylogenetic tree analysis was conducted using KSNP3.0 with a bootstrap of 1,000, and a minimum spanning tree was constructed based on the scaffold sequence. The geographic distribution of clustered strains was mapped using Python. Raw whole-genome sequencing data were deposited in the National Library of Medicine under project number PRJNA1044236.

### Definitions

Treatment-naïve tuberculosis patients: Individuals meeting any of the following criteria: (i) patients with tuberculosis who have not previously received anti-tuberculosis medications; (ii) tuberculosis patients with an inconsistent treatment duration of less than 1 month; and (iii) patients undergoing standardized treatment who have not completed the treatment course.

Patients with re-treated tuberculosis: Individuals meeting any of the following criteria: (i) patients who have experienced treatment failure or recurrent tuberculosis and (ii) patients whose anti-tuberculosis treatment duration has been irregular and exceeded 1 month.

Multidrug-resistant TB: It is defined as TB in which the causal *Mycobacterium tuberculosis* bacteria are resistant to both rifampicin and isoniazid, regardless of its resistance to other anti-TB drugs.

Pre-XDR-TB: The condition in which *Mycobacterium tuberculosis* demonstrates resistance to either at least one fluoroquinolone in the context of MDR-TB/RR-TB.

Extensively drug-resistant TB: MDR/RR-TB that demonstrates resistance to a fluoroquinolone and at least one other group A drug, such as bedaquiline or linezolid.

Diagnostic delay: The interval from the onset of symptoms to the date of tuberculosis diagnosis spanning more than 2 months.

Clusters: Two isolates exhibiting a genetic distance of less than 12 SNPs.

### Statistical analysis

The initial data set was created using Microsoft Excel 2022, and IBM SPSS v26.0 was used for statistical analysis. The demographic and clinical features of patients were compared using chi-square tests, corrected chi-square tests, or Fisher’s exact probability method. The recent spread of *Mycobacterium tuberculosis* was assessed for influencing factors through initial screening using univariate regression analysis. Factors with a *P*-value <0.05 were included in the multivariate logistic regression model analysis to identify independent risk factors. A *P*-value <0.05 was considered to be statistically significant.

## Data Availability

Data of this study are fully available at https://www.ncbi.nlm.nih.gov/bioproject/1044236 and without restriction upon reasonable request.

## SUPPLEMENTAL MATERIAL

Table S1 spectrum.03555-23-s0001.docx Critical concentration value of anti-tuberculosis drugs by liquid culture.

ASM does not own the copyrights to Supplemental Material that may be linked to, or accessed through, an article. The authors have granted ASM a non-exclusive, world-wide license to publish the Supplemental Material files. Please contact the corresponding author directly for reuse.
