# Supplementary material for: Transmission of multidrug-resistant tuberculosis in Jiangxi, China, and associated risk factors
Source: Microbiol Spectr. 2024 Oct 2;12(11):e03555-23. doi: 10.1128/spectrum.03555-23 (PMC11537056; doi:10.1128/spectrum.03555-23)
Supplement: Table S1 — Critical concentration value of anti-tuberculosis drugs by liquid culture. [file spectrum.03555-23-s0001.docx]

Addition Table 1：Critical concentration value of anti-tuberculosis drugs by liquid culture

| Drug | English abbreviation | Critical concentration value（μg/ml） |
| --- | --- | --- |
| Isoniazid | INH | 0.1 |
| Rifampicin | RIF | 1.0 |
| Streptomycin | SM | 1.0 |
| Ethambutol | EMB | 5.0 |
| Amikacin | AM | 1.0 |
| Capreomycin | CM | 2.5 |
| Levofloxacin | LEV | 2.0 |
| Prothionamide | PTO | 2.5 |
| Clarithromycin | CLA | 1.0 |
| Cycloserine | CS | 10.0 |
| Moxifloxaci | MFX | 0.25 |
